# Direct comparison of efficacy of combining ivermectin versus five first-line chemotherapy drugs with recombinant methioninase against colon-cancer cells

**DOI:** 10.3389/fonc.2026.1807785

**Published:** 2026-06-03

**Authors:** Jinsoo Kim, Qinghong Han, Shukuan Li, Byung Mo Kang, Yuta Miyashi, Tomoyuki Ishiguro, Michael Bouvet, Robert M. Hoffman

**Affiliations:** 1 AntiCancer Inc., San Diego, CA, United States; 2 Department of Surgery, University of California, San Diego, San Diego, CA, United States; 3 Department of Surgery, Chungnam National University College of Medicine, Daejeon, Republic of Korea

**Keywords:** first-line chemotherapy, ivermectin, recombinant methioninase, combination, efficacy, HCT116 colon-cancer cells

## Abstract

**Background/aim:**

Ivermectin is emerging as a potential anticancer agent. Recombinant methioninase (rMETase) targets methionine addiction of cancer and shows synergistic efficacy when combined with cancer-chemotherapy drugs. In the present study, we compared the efficacy of the rMETase combination with ivermectin versus rMETase combined with each of five first-line chemotherapeutic agents, on HCT116 human colon-cancer cells.

**Materials and methods:**

The half-maximal inhibitory concentrations (IC_50_) of rMETase, ivermectin, and five first-line chemotherapy drugs were determined from dose-response curves. HCT116 colon-cancer cells were then treated with each drug at its IC_50_ concentration, either alone, or in combination with rMETase, at their IC_50_ concentration. Cell viability was assessed after 72 h using the WST-8 assay. A chemosensitivity index (CI) was defined as the ratio of cancer-cell viability after treatment with each drug alone at its IC_50_ to that after treatment with the same drug at its IC_50_ concentration combined with rMETase.

**Results:**

At the IC_50_ concentration, ivermectin combined with rMETase (CI, 6.7 ± 1.9) was significantly (p < 0.05) more effective than rMETase combined with 5-fluorouracil (CI, 2.0 ± 0.7); cisplatinum (CI, 2.4 ± 1.5); gemcitabine (CI, 2.5 ± 0.7); and paclitaxel (CI, 2.8 ± 0.5). Only doxorubicin combined with rMETase was slightly more effective than rMETase combined with ivermectin (CI, 7.8 ± 1.7).

**Discussion:**

Ivermectin combined with rMETase was more effective than four of five first-line chemotherapy drugs combined with rMETase against colon-cancer cells, demonstrating additional promise of ivermectin as an anticancer drug.

## 1 Introduction

Ivermectin is emerging as a potential anticancer agent, but its effectiveness depends on combination with other agents (1–3). Methionine addiction is a fundamental and general metabolic hallmark of cancer, known as the Hoffman effect (4–10). Methionine addiction is targeted in cancer by methionine restriction, including recombinant methioninase (rMETase) (11–14).

Previous studies have shown synergistic efficacy of rMETase in combination with numerous chemotherapies against cancer *in vitro* and *in vivo* (15–22). *In vitro*, ivermectin combined with rMETase has showed synergistic efficacy (23–25) and greater cytotoxicity on cancer cells than on normal cells (25). However, a systematic head-to-head comparison of ivermectin and multiple chemotherapeutic agents, each in combination with rMETase, has not been performed in the same cancer cell line under standardized conditions. In the present study, we compared the efficacy of combining rMETase plus ivermectin with that of rMETase combined with each of five first-line chemotherapy agents on cancer cells *in vitro.*

## 2 Materials and methods

### 2.1 Cell culture

HCT116 human colon-cancer cells were obtained from the American Type Culture Collection (Manassas, VA, USA). The cells were maintained in Dulbecco’s Modified Eagle’s Medium/Nutrient Mixture F-12 with GlutaMAX™ supplement (DMEM/F-12; Thermo Fisher Scientific, Waltham, MA, USA) supplemented with 10% fetal bovine serum (FBS) and 1% penicillin/streptomycin. Cells were cultured at 37 °C in a humidified atmosphere containing 5% CO_2_ and were passaged at 70–80% confluence.

### 2.2 Recombinant methioninase production

rMETase was produced by AntiCancer Inc. (San Diego, CA, USA) via fermentation of recombinant *Escherichia coli* transformed with the *methioninase* gene from *Pseudomonas putida*. The purification process involved heat treatment at 60 °C, polyethylene glycol precipitation, and diethylaminoethyl (DEAE) Sepharose fast-flow ion-exchange column chromatography, as previously described (11).

### 2.3 Reagents

Ivermectin was purchased from MedChemExpress (Monmouth Junction, NJ, USA) and dissolved in dimethyl sulfoxide (DMSO) to prepare a 10 mM stock solution. Doxorubicin was obtained from Dr. Reddy’s Laboratories, Ltd. (Hyderabad, Telangana, India), and paclitaxel was obtained from Fresenius Kabi USA, LLC (Lake Zurich, IL, USA). Cisplatinum was obtained from WG Critical Care LLC (Paramus, NJ, USA); 5-fluorouracil was obtained from Sandoz Inc. (Princeton, NJ, USA); gemcitabine was obtained from BluePoint Laboratories (Little Island, Cork, Ireland). Each drug was diluted in 0.9% normal saline to prepare stock 10 mM solutions which was then diluted in DMEM/F-12 cell-culture medium and added to cell-culture wells at the desired concentrations.

### 2.4 Determination of half-maximal inhibitory concentration (IC_50_) values

Dose–response curves for rMETase and each chemotherapeutic agent were generated using a colorimetric cell-viability assay as follows: HCT116 cells were seeded at 2.0 × 10³ cells per well in 96-well plates and allowed to adhere overnight. Cells were then treated for 72 h with serial dilutions of rMETase or each drug over a wide concentration range. After treatment, cell viability was assessed using the Cell Counting Kit-8 (Dojindo Laboratories, Kumamoto, Japan) containing the WST-8 cell-viability reagent. Ten microliters of WST-8 solution were added to each cell-culture well, followed by a 1 h incubation at 37 °C. Absorbance was then measured at 450 nm using a microplate reader (Sunrise; Tecan, Männedorf, Switzerland). Cell viability was calculated as a percentage relative to untreated control cells. Microsoft 365 Excel for MacOS (Microsoft, Redmond, WA, USA) was used for raw data organization. ImageJ version 1.54g (National Institutes of Health, Bethesda, MD, USA) was used only for image-based quantification, and not for direct calculation of IC_50_ values. Dose–response curves and IC_50_ values were generated using GraphPad Prism version 10.6.1 (GraphPad Software, Inc., San Diego, CA, USA) by fitting the data to a four-parameter logistic model with nonlinear regression. Data are presented as mean ± standard deviation.

### 2.5 Treatment with each drug alone or combined with rMETase at their IC_50_ concentrations

To compare the efficacy of rMETase combined with ivermectin versus combination with each of five first-line chemotherapy agents under standardized conditions, HCT116 cells were seeded in 96-well plates at a density of 2.0 × 10^3^ cells/well in DMEM/F-12 and allowed to adhere overnight. HCT116 cells were then treated with ivermectin, doxorubicin, 5-fluorouracil, cisplatinum, gemcitabine, or paclitaxel at their respective IC_50_ concentrations, either alone, or in combination with rMETase at their IC_50_ concentration for 72 h. Untreated cells served as controls. Each experimental condition was tested in multiple replicate wells. The experiment were repeated six independent times. Data are presented as the mean ± standard deviation (SD) from six independent experiments (n = 6).

### 2.6 Calculation of a chemosensitivity index

To quantitatively compare the efficacy of rMETase combined with either ivermectin or each of the first-line chemotherapeutic agents, we calculated a chemosensitivity index (CI) for each drug. CI was defined as the ratio of cell viability after treatment with each chemotherapeutic agent alone at its IC_50_ concentration to that after treatment with the same drug at its IC_50_ combined with rMETase at its IC_50_ concentration: CI = (viability with drug alone at IC_50_)/(viability with drug at IC_50_ + rMETase at IC_50_). A higher CI indicates stronger efficacy of the drug combined with rMETase.

### 2.7 Statistical analysis

Dose–response curves were fitted using a four-parameter logistic model with nonlinear regression. Data are presented as mean ± standard deviation. Comparisons between groups were performed using one-way analysis of variance (ANOVA). Statistical significance was determined using Dunnett’s multiple comparison test. A p-value ≤ 0.05 was considered statistically significant.

## 3 Results

### 3.1 Determination of IC_50_ values of rMETase and ivermectin and five first-line chemotherapeutic agents on HCT116 cells

**Figure 1:**
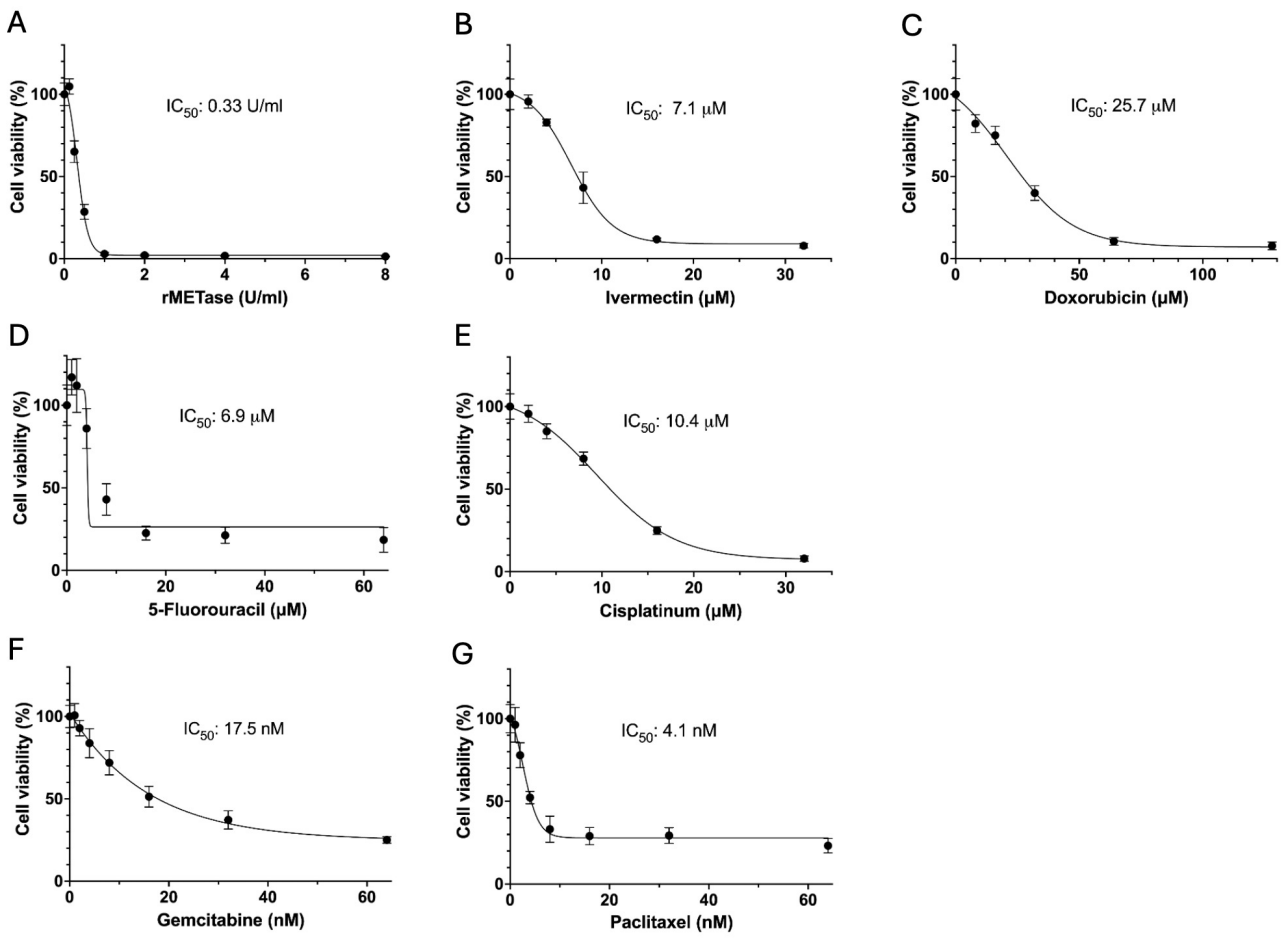
Dose–response curves and IC_50_ values of recombinant methioninase (rMETase), ivermectin, and five first-line chemotherapeutic agents on HCT116 colon-cancer cells. Cells were treated for 72 h with increasing concentrations of **(A)** rMETase; **(B)** ivermectin; **(C)** doxorubicin; **(D)** 5-fluorouracil; **(E)** cisplatinum; **(F)** gemcitabine; or **(G)** paclitaxel. Please see Materials and methods for details. Data are expressed as mean ± SD.

Dose–response curves for rMETase, ivermectin, and each first-line chemotherapeutic agent are shown in **Figure 1**. rMETase alone had an IC_50_ of 0.33 U/ml on HCT116 cells. The IC_50_ values on HCT116 cells were 7.1 μM for ivermectin; 25.7 μM for doxorubicin; 6.9 μM for 5-fluorouracil; 10.4 μM for cisplatinum; 17.5 nM for gemcitabine; and 4.1 nM for paclitaxel (**Figure 1**). The IC_50_ value of doxorubicin on HCT116 cells in the present study differs from previously published values (26, 27) and depends on specific experimental conditions. These IC_50_ values were used as the fixed drug concentrations for subsequent combination experiments.

### 3.2 Cytotoxicity of rMETase combined with ivermectin and each of five first-line chemotherapeutic agents at their IC_50_ concentrations on HCT116 cells

**Figure 2:**
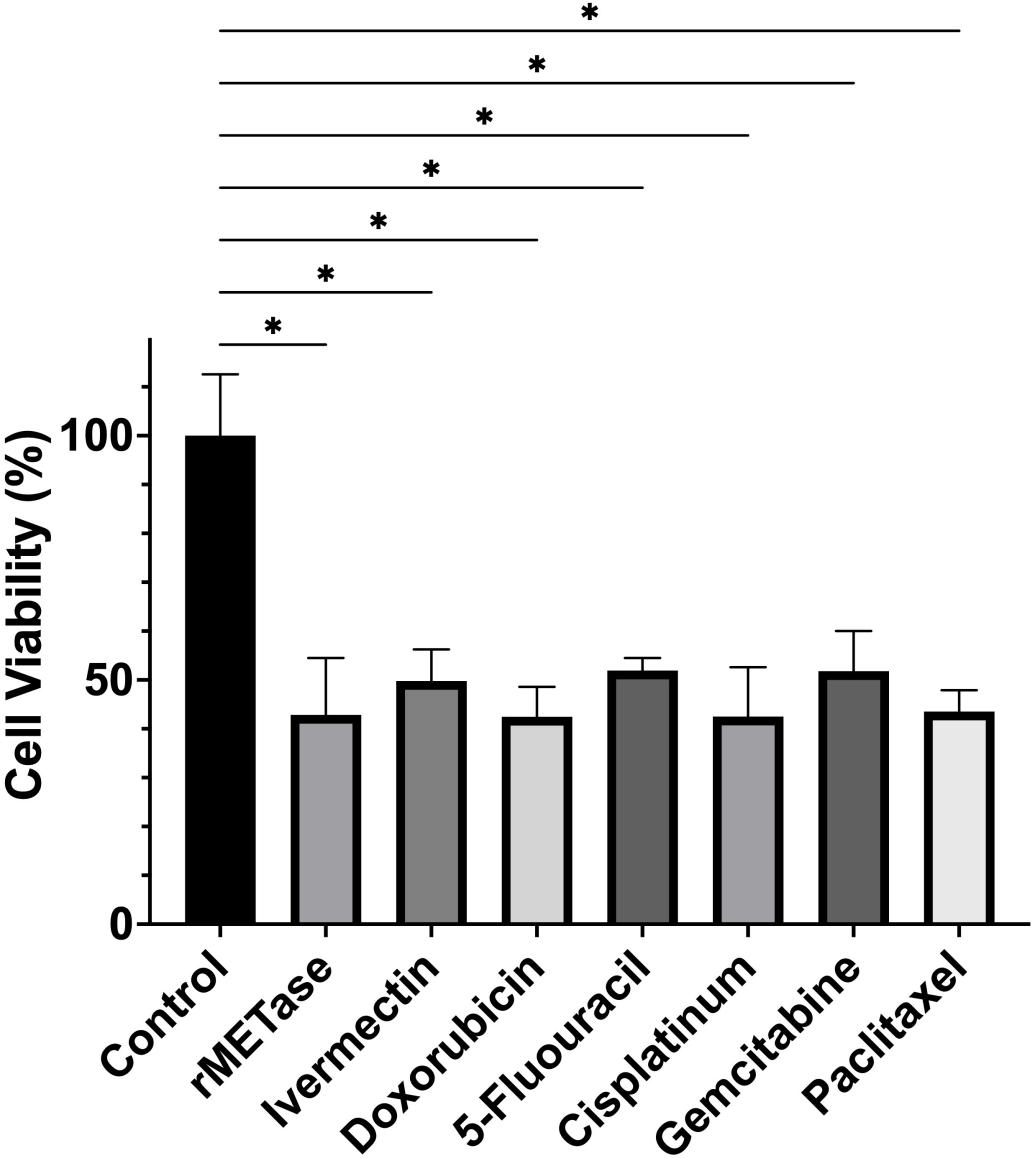
Comparison of cytotoxicity of recombinant methioninase (rMETase), ivermectin and five first-line chemotherapeutic agents at their IC_50_ concentrations on HCT116 colon cancer cells. Please see Materials and methods for details. Data are mean ± SD. *p<0.05 versus control.

Treatment with rMETase and each drug alone at its IC_50_ for 72 h significantly reduced cell viability compared with untreated control cells (**Figure 2**). HCT116 cell viability reduction rates compared to control were 57.1 ± 11.6% for rMETase; 50.2 ± 6.4% for ivermectin; 57.3 ± 6.1% for doxorubicin; 48.1 ± 2.5% for 5-fluorouracil; 57.5 ± 10.1% for cisplatinum; 48.2 ± 8.2% for gemcitabine, and 56.4 ± 4.3% for paclitaxel.

### 3.3 Efficacy of ivermectin or each of five first-line chemotherapeutic agents combined with rMETase on HCT116 colon-cancer cells

**Figure 3:**
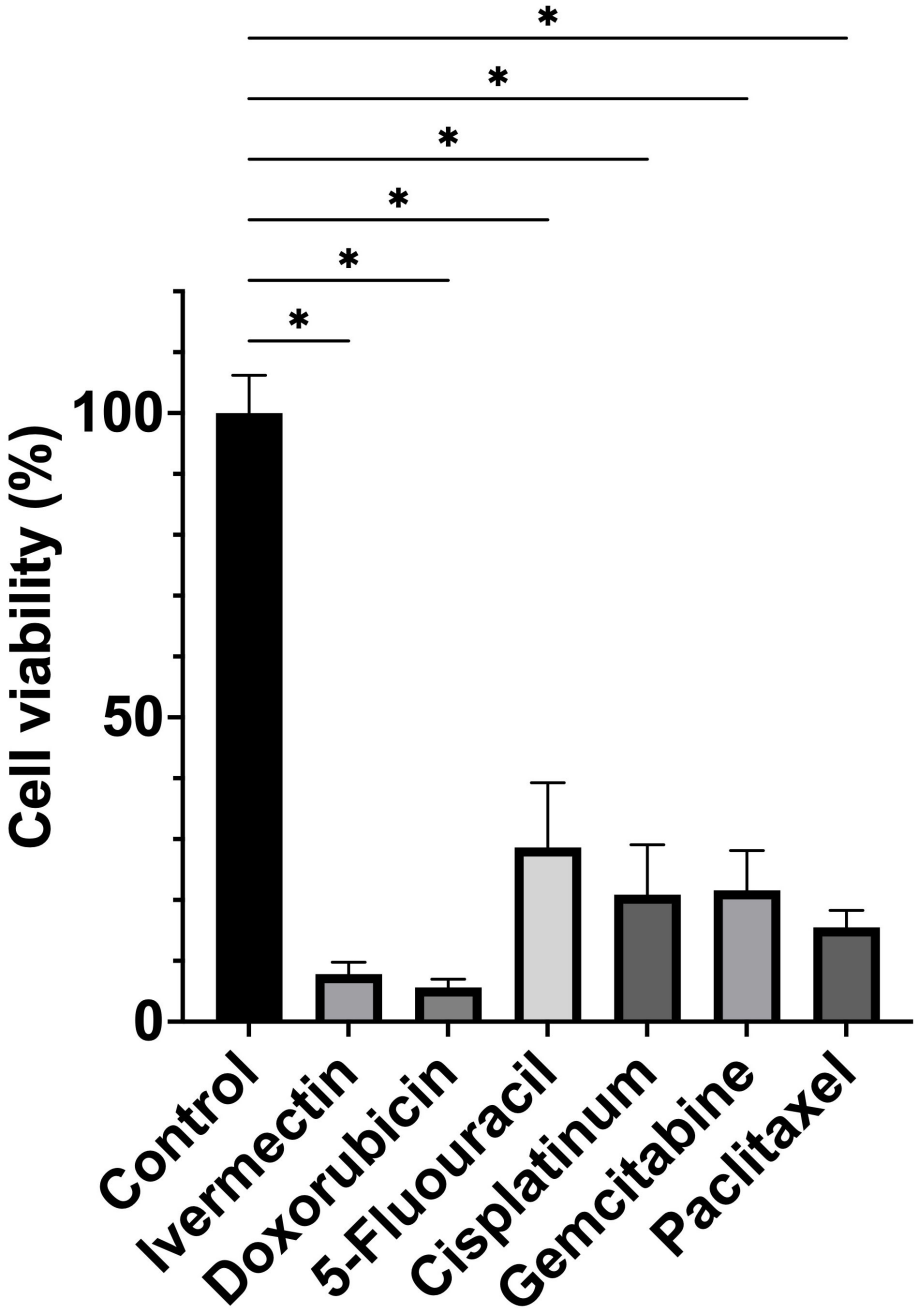
Comparison of efficacy of recombinant methioninase (rMETase) combined with ivermectin versus each of five first-line chemotherapeutic agents combined with rMETase, at their IC_50_ concentrations, on HCT116 cell viability. Please see Materials and methods for details. Data are mean ± SD. *p<0.05 versus the corresponding drug-alone group.

The combination of ivermectin with rMETase (viability 7.8 ± 1.9% of control) was more effective than rMETase combined with 5-fluorouracil (viability 28.5 ± 10.3% of control); cisplatinum (viability 20.8 ± 8.1% of control); gemcitabine (viability 21.5 ± 6.5% of control); paclitaxel (viability 15.5 ± 2.7% of control); and was comparable to doxorubicin (viability 5.6 ± 1.3% of control) (**Figure 3**).

### 3.4 CI of rMETase combined with ivermectin versus rMETase combined with each of five first-line chemotherapeutic agents

**Figure 4:**
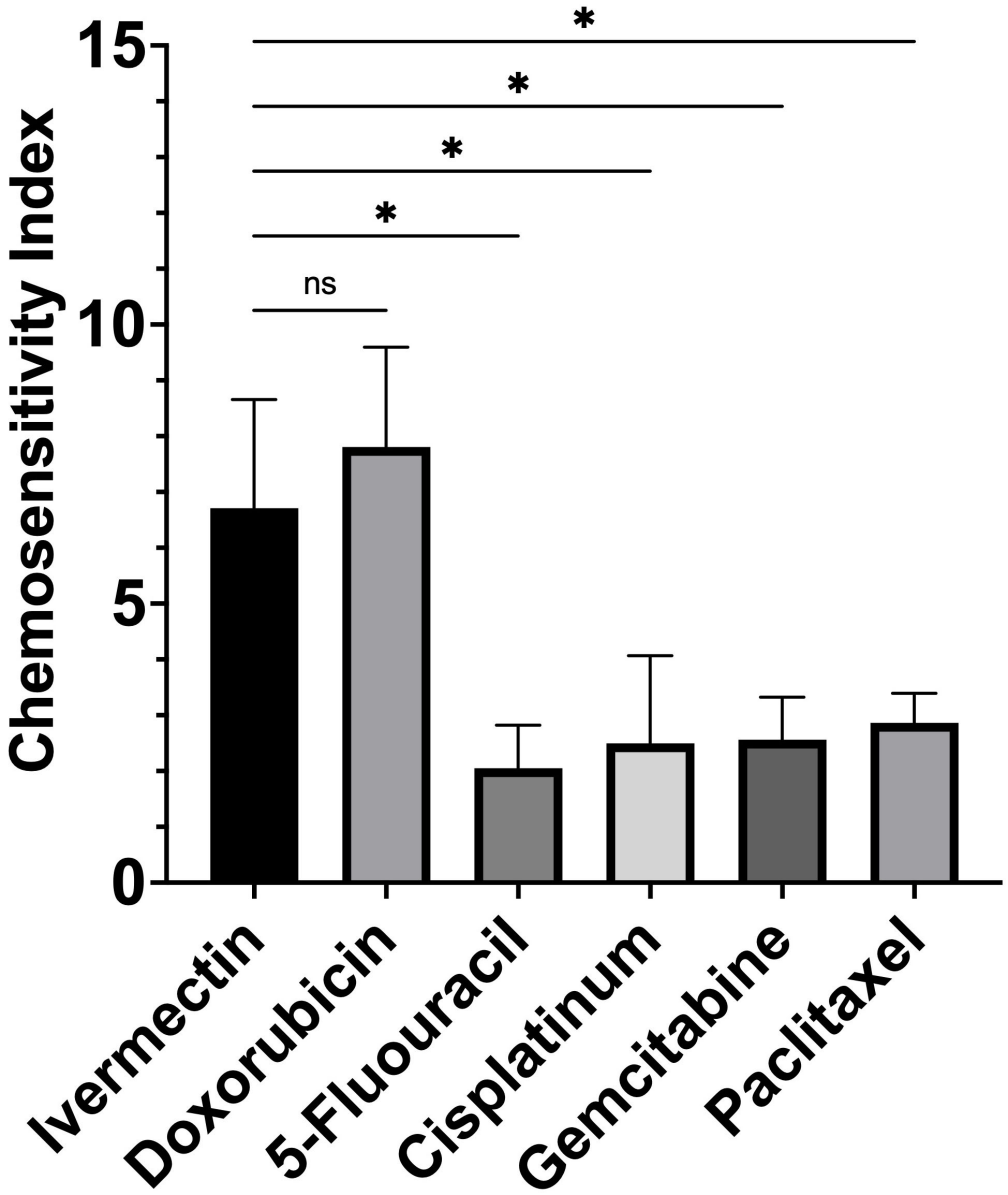
Comparison of the chemosensitivity index of recombinant methioninase (rMETase) in combination with ivermectin versus five first-line chemotherapeutic agents combined with rMETase, on HCT116 colon cancer cells. The chemosensitivity index was defined as the ratio of cell viability after treatment with each drug alone at its IC_50_ concentration to that after combination treatment with the same drug combined with rMETase at their IC_50_ concentration. Please see Materials and methods for details. Bars represent mean ± SD of chemosensitivity index values from six independent experiments. Ivermectin or doxorubicin combined with rMETase showed significantly higher chemosensitivity index values than 5-fluorouracil, cisplatinum, gemcitabine or paclitaxel combined with rMETase (*p<0.05, ns, non-specific).

CI values were 2.0 ± 0.7 for 5-fluorouracil plus rMETase; 2.4 ± 1.5 for cisplatinum plus rMETase; 2.5 ± 0.7 for gemcitabine plus rMETase and 2.8 ± 0.5 for paclitaxel plus rMETase, indicating an approximately two- to three-fold enhancement of the cytotoxicity of each drug by rMETase. In contrast, ivermectin and doxorubicin, each combined with rMETase, had CI values of 6.7 ± 1.9 and 7.8 ± 1.7, respectively (**Figure 4**). The CI value for ivermectin combined with rMETase was significantly higher than those for 5-fluorouracil, cisplatinum, gemcitabine and paclitaxel, each combined with rMETase (p < 0.05), whereas no significant difference was observed between ivermectin and doxorubicin, each combined with rMETase.

## 4 Discussion

The main finding of the present study is that ivermectin combined with rMETase was more effective against colon-cancer cells than rMETase combined with major first-line chemotherapeutic agents, including 5-fluorouracil, cisplatinum, gemcitabine, and paclitaxel, except for doxorubicin. Ivermectin and doxorubicin yielded the highest CI value and approximately three-fold greater than those of 5-fluorouracil, cisplatinum, gemcitabine, and paclitaxel.

However, in contrast to doxorubicin combined with rMETase, we previously showed ivermectin combined with rMETase has limited toxicity against normal cells (24).

The synergy between ivermectin and rMETase may be related, at least in part, to their effects on cell-cycle progression, as rMETase traps cancer cells in the S/G2 phase and ivermectin induces S-phase arrest in colon-cancer cells (28, 29).

The present study has several limitations, including its *in vitro* design and the use of a single cancer cell line, HCT116, which was selected because ivermectin and rMETase had previously shown strong synergy in this model. Nonetheless, the present results indicate that the combination of rMETase and ivermectin is promising. Future studies are warranted to evaluate this combination in mouse cancer models and, ultimately, in clinical trials.

## Data Availability

The original contributions presented in the study are included in the article/supplementary material. Further inquiries can be directed to the corresponding author.
